# Examining Different Motor Learning Paradigms for Improving Balance Recovery Abilities Among Older Adults, Random vs. Block Training—Study Protocol of a Randomized Non-inferiority Controlled Trial

**DOI:** 10.3389/fnhum.2021.624492

**Published:** 2021-02-25

**Authors:** Hadas Nachmani, Inbal Paran, Moti Salti, Ilan Shelef, Itshak Melzer

**Affiliations:** ^1^Department of Physical Therapy, Faculty of Health Sciences, Ben-Gurion University of the Negev, Beer-Sheva, Israel; ^2^Brain Research Imaging Center, Ben-Gurion University of the Negev, Beer-Sheva, Israel; ^3^Diagnostic Imaging Department, Soroka University Medical Center, Beer-Sheva, Israel

**Keywords:** elderly people, postural balance, falls, balance perturbation training, random training, block training

## Abstract

**Introduction**: Falls are the leading cause of fatal and nonfatal injuries among older adults. Studies showed that older adults can reduce the risk of falls after participation in an unexpected perturbation-based balance training (PBBT), a relatively novel approach that challenged reactive balance control. This study aims to investigate the effect of the practice schedule (i.e., contextual interference) on reactive balance function and its transfer to proactive balance function (i.e., voluntary step execution test and Berg balance test). Our primary hypothesis is that improvements in reactive balance control following block PBBT will be not inferior to the improvements following random PBBT.

**Methods and Analysis**: This is a double-blind randomized controlled trial. Fifty community-dwelling older adults (over 70 years) will be recruited and randomly allocated to a random PBBT group (*n* = 25) or a block PBBT group (*n* = 25). The random PBBT group will receive eight training sessions over 4 weeks that include unexpected machine-induced perturbations of balance during hands-free treadmill walking. The block PBBT group will be trained by the same perturbation treadmill system, but only one direction will be trained in each training session, and the direction of the external perturbations will be announced. Both PBBT groups (random PBBT and block PBBT) will receive a similar perturbation intensity during training (which will be customized to participant’s abilities), the same training period, and the same concurrent cognitive tasks during training. The generalization and transfer of learning effects will be measured by assessing the reactive and proactive balance control during standing and walking before and after 1 month of PBBT, for example, step and multiple steps and fall thresholds, Berg balance test, and fear of falls. The dependent variable will be rank transformed prior to conducting the analysis of covariance (ANCOVA) to allow for nonparametric analysis.

**Discussion**: This research will explore which of the balance retraining paradigms is more effective to improve reactive balance and proactive balance control in older adults (random PBBT vs. block PBBT) over 1 month. The research will address key issues concerning balance retraining: older adults’ neuromotor capacities to optimize training responses and their applicability to real-life challenges.

**Clinical Trial Registration**: Helsinki research ethics approval has been received (Soroka Medical Center approval #0396-16-SOR; MOH_2018-07-22_003536; www.ClinicalTrials.gov, NCT04455607).

## Background

One out of four older adults will fall each year (Bergen et al., 2016), and the medical cost in 2015 for fatal and nonfatal fall-related injuries was 50 billion dollars (Centers for Disease Control and Prevention, 2017; Florence et al., 2018). The psychological impact of a fall often results in increasing self-restriction of activities and a decrease in quality of life (King and Tinetti, 1996; Rubenstein, 2006; Bergen et al., 2016).

Most fall prevention exercise programs incorporate balance practice which involves volitional movements (Gillespie et al., 2003, 2012; Lord et al., 2003; Wolf et al., 2003; Hue et al., 2004; Sihvonen et al., 2004; Skelton et al., 2005; Liu-Ambrose et al., 2008; Clemson et al., 2012). In a systematic review that included 81 trials (*n* = 19,684), Sherrington et al. (2019) found that training programs that included voluntary exercises (e.g., Otago program, FAME, and tai chi) reduce the rate of falls by 23% [relative risk (RR) 0.77; confidence interval (CI) 0.71, 0.83] and reduce the rate of fallers by 15% (RR 0.85; CI 0.81, 0.89). However, it is believed that balance recovery strategies that are evoked by an external unexpected loss of balance, i.e., unexpected perturbations, *cannot* be trained through voluntary exercises. In view of the evidence that the neural balance control differs in some fundamental ways in comparison to balance reactions that are evoked unexpectedly when balance is lost, it can be argued that training approaches for preventing falls should involve the use of unexpected perturbations during training (Maki et al., 2008). There is now accumulating evidence that shows the superiority of balance training that included unexpected perturbation exercises. Among older adults, just 24 perturbations within a single session of perturbation-based balance training (PBBT) is sufficient to lead to lasting improvements (i.e., 6–12 months) in reactive balance control (Bhatt et al., 2012) and prevent falls in daily life (Pai et al., 2014a). In a systematic review, it was found that older adults who participated in a PBBT that challenged the mechanisms responsible for dynamic stability could adapt in a reactive manner (Mansfield et al., 2015). Furthermore, they showed a reduction in the rate of falls by 46% and the diverse risks of falls (Mansfield et al., 2015).

Several studies have evaluated perturbation-based training during walking in older adults (Shimada et al., 2004; Mansfield et al., 2010; Halvarsson et al., 2011; Grabiner et al., 2012; Lurie et al., 2013; Pai et al., 2014a; Kurz et al., 2016; Okubo et al., 2017; McCrum et al., 2017). In another systematic review (Gerards et al., 2017), the authors reported that the PBBT that incorporates multiple perturbation types and directions might be of most benefit to improve balance. Studies reported that PBBT was accepted by older adults (Shimada et al., 2004; Mansfield et al., 2007; Melzer et al., 2007b; Pai and Bhatt, 2007; Lurie et al., 2013) and reported improvements in balance performance even after a single training session (Pai et al., 2014a). All the above training programs did not explore the skill acquisition of random practice compared to block practice in terms of the directions of perturbations of unexpected PBBT for older adults.

The process of skill acquisition relies on the interaction between cognition and motor control. One demonstration of this interaction has been termed the contextual interference (CI) effect (Lee and Magill, 1983). For example, practice schedule, the influence of the order in which training materials are presented to the learner, may influence learning abilities (Jamieson and Rogers, 2000). In training programs, the practice schedule is mainly divided into blocked or random practice. We define blocked practice as practicing a single task repeatedly before moving on to the next task (for example, train only unexpected loss of balance to the right direction, i.e., right balance reactive stepping, in a specific training session). Random practice is defined as practicing the tasks in a pseudo-random order (Shea and Morgan, 1979) such that each task is not practiced consecutively (for example, train balance recovery, including reactive stepping to the right/left/forward/backward randomly at the same training session). There is evidence that suggests that random training can improve motor learning [from pressing a button in response to a light cue, automated teller machine (ATM) operation, hand grasp as a response to a stimulus and knocking down six (changing) barriers, isometric pinch force to perturbation training, et cetera (Shea and Morgan, 1979; Del Rey, 1982; Kausler et al., 1990; Jamieson and Rogers, 2000; Guadagnoli and Lee, 2004; Bhatt and Pai, 2009; Mansfield et al., 2010; Hurt et al., 2011; Bhatt et al., 2012; Fazeli et al., 2017)]; on the other hand, others suggest that block training (cognitive memory task, balance perturbation training, seat to stand training, and isometric pinch force control) is more effective (Del Rey, 1982; Lazarus and Haynes, 1997; Pavol et al., 2002, 2004; Lin et al., 2008; Van Ooteghem et al., 2009; Pai et al., 2014a, b; Dijkstra et al., 2015; Coelho and Teixeira, 2017, 2018; Coelho et al., 2018). Additionally, a high CI effect (i.e., random practice) may create a poorer performance acquisition but enhance learning and better retention or transfer performance. Another research (Wright et al., 2016) has shown that in high CI, brain areas that are functionally significant for motor learning are recruited earlier. Lage et al. (2015) studied the correlation between different practice schedules and engagement of cortical brain areas. In their review that included 10 studies of different motor skill acquisitions, they found that there is greater activation of neural structures during random practice than during block practice. Additionally, random practice had a greater involvement of cognitive processes. On the other hand, de Xivry and Lefèvre (2015) found that different perturbation schedules did not lead to a more or less stabilized motor memory. A recent systematic review (Graser et al., 2019) of 25 studies examining the role of the practice order in children showed limited evidence for the benefit of blocked practice over random practice in regard to acquisition and retention; only for transfer there is moderate consistent evidence for the benefit of random practice over blocked practice. An additional approach of perturbation training is utilizing a split-belt training paradigm in which a person could walk on a treadmill with two belts moving at different speeds. Shimada et al. (2004) showed a significant improvement in balance function and a reduction in the number of falls (21% lower) in the split treadmill exercise. However, most falls are the result of unexpected perturbation, such as stumbling or slipping while walking, and not the result of walking on a different walking speed (Gabell et al., 1985).

Given the importance of balance recovery including reactive stepping in avoiding falls and the fact that balance loss is always unexpected and multidirectional, it is important to explore which PBBT program (random PBBT vs. block PBBT) is more effective or noninferior for balance recovery skill acquisition. To our knowledge, only a handful of studies (Pavol et al., 2002, 2004; Bhatt and Pai, 2009; Van Ooteghem et al., 2009; Hurt et al., 2011; Bhatt et al., 2012; Pai et al., 2014a, b; Dijkstra et al., 2015; Coelho and Teixeira, 2017, 2018; Coelho et al., 2018) published to date used some principles of motor learning theory to achieve the maximum skill acquisition, retention, and generalization of these learned skills. Unlike other forms of exercise, improved reactive balance control with PBBT seems to occur with few repetitions (McCrum et al., 2017) and is preserved for several months after training (McCrum et al., 2017).

From a fall-prevention standpoint, an important aim of a PBBT is that learned motor skills (i.e., reactive balance performance) create a fast learning process, which can be generalized to a wide variety of everyday situations (i.e., real-life situations), which may require rapid balance recovery maneuvers. In general, motor learning research suggests that conditions during training should be varied randomly to optimize motor learning [challenge point framework (CPF; Guadagnoli and Lee, 2004)]. This would mean delivering perturbations from various directions in a randomized fashion during PBBT and different phases of the gait cycle. To our knowledge, no study has investigated whether a constant or random practice is more effective for motor skill acquisition, retention, and transferring motor tasks when older adults are training to improve their ability to recover from an unexpected loss of balance during walking. Our proposed study will incorporate similar principles with the use of a mechatronic device that can perturb participants unexpectedly and in multidirectional ways during treadmill walking. It is not known whether random PBBT better improves balance reactions in terms of the period of PBBT than block PBBT. In this research, we will address a key question about the generalizability of balance intervention and the underlying locomotor plasticity in older adults using random vs. block PBBT. The primary aim of this study is to determine the effect of block PBBT on reactive balance control among older adults (i.e., multiple- and fall-step thresholds). Our primary hypothesis is that improvements in reactive balance control following block PBBT will be not inferior to the improvements following random PBBT. The secondary purpose of this study is to determine the effect of block PBBT and random PBBT on balance proactive balance control and fear of falls. We hypothesize that, proactive balance, i.e., anticipatory balance control [Berg Balance Scale (BBS)] and fear of fall (FES-I), will cause similar effects in block PBBT and random PBBT.

## Methods and Analysis

### Study Design and Setting

**Figure 1:**
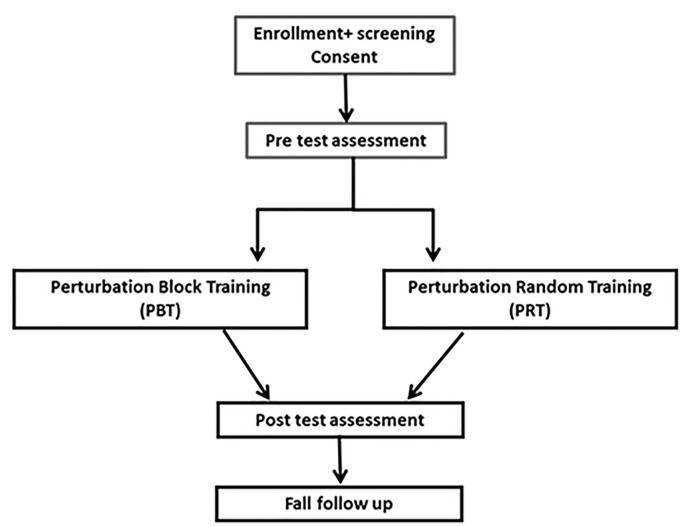
Study flowchart.

This is a double-blind randomized controlled trial (Figure 1, flowchart) that follows the recommendations of SPIRIT 2013 (see Supplementary Appendix 1 for the SPIRIT study checklist). Older adults are randomly assigned to one of two groups: (1) random PBBT; and (2) block PBBT. Both groups are trained twice a week for 4 weeks. Compensatory (reactive) and anticipatory (proactive) balance control during standing and walking, functional balance, and fear of falling will be measured pre-training and post-training. Fall monitoring will be performed 6 months after the posttest (Table 1). The PBBT will be provided inside participants’ community centers or in their protected housing. The study was approved by the Helsinki ethics committee at Soroka Medical Center, Beer-Sheva, Israel [Soroka Medical Center approval #0396-16-SOR; Israeli Ministry of Health (MOH)_#2018-07-22_003536; www.ClinicalTrials.gov, NCT04455607].

**Table 1:** Flowchart—overview of outcome measurements and time of assessment.

	T_0_	T_1_	T_T_	T_2_	T_3_
Recruitment	+				
Interview (in/exclusion)	+				
Consent form (and medical waiver)		+			
Balance assessment (balance reactions while standing and walking; postural stability)		+		+	
Randomization		+			
Questionnaires (BBS; MMSE; FES-1)		+		+	
Training documentation (perturbation progression; participant comments; adverse events)			+		
Fall monitoring					+
Satisfaction questionnaire				+	

*T_0_, prior to study; T_1_, baseline (pre-intervention); T_T_, training time; T_2_, post-intervention test; T_3_, follow-up (6 months post-intervention). Abbreviations: BBS, Berg Balance Scale; MMSE, Mini-Mental Scale Examination; FES-1, fear of fall questionnaire*.

### Participants

A convenient sample of 50 community-dwelling older adults will be recruited from retirement villages for older adults *via* advertisements, personal contacts, and word of mouth. Participants will be included in the study if they are 70 years or older and independent in daily living activities and walking without assistive devices. After completing medical history, volunteers will be excluded if one met the exclusion criteria [suffering from ischemic heart disease which limits exercise; suffering from COPD and uncontrolled blood pressure; suffering from serious vision problems; having a score under 24 on the Mini-Mental State Examination (MMSE); being under a year after hip or knee replacement or broken extremities; and having any neurological diseases or stroke]. All subjects will provide a medical waiver signed by their primary care physician clearing them to participate in moderate physical exercise. All subjects will sign an informed consent statement. Participants will be informed that this is an intervention method of a technology that aimed to improve balance while treadmill walking and that based on the literature, we hypothesize that they will be most likely improving their function. They will also be informed that the training might be difficult at the beginning and thus muscle soreness will occur and that they are free to withdraw from the study at any time point, without consequence. They may also be withdrawn from the study due to changes in their health status that affect eligibility.

### Recruitment, Randomization, Blinding, and Treatment Allocation

Participants will be reimbursed with $25 for travel expenses (e.g., public transit, taxi, or parking) they incur to attend data collection appointments. Participants will be assigned using randomization to one of the two groups. The random allocation sequence will be computer generated (Random Allocation Software version 1.0). Blocked randomization will ensure equal numbers allocated to each group. Group allocation will be performed centrally by the principal investigator, who will not be involved in recruiting, scoring assessments, or administering the interventions (i.e., concealed allocation). Outcome measures will be obtained by a research assistant who will be blinded to group allocation. Participants are also blinded to group allocation since they will train on the same mechatronic device.

### Informed Consent

In case an older adult is willing to participate in the study, a researcher (HN) will explain the study and will provide the participant with the study information sheet, information form for the general practitioner, and their permission to participate in the study and consent form. Participants will be informed that in this study there are two types of interventions that expose the participants to perturbations during treadmill walking (random PBBT vs. block PBBT); both are expected to improve balance function. To maintain the motivation of older adults to participate, they will be offered to participate in “other” exercise programs after the training period. They are also informed that the training might be difficult in the beginning and thus some muscle soreness will occur and that participants are free to withdraw from the study at any time point, without consequence. Participants may also be withdrawn from the study due to changes in their health status that affect eligibility. HN will answer the participant questions about the study. Participants may discuss the study with their family members, friends, or healthcare providers. The informed consent process will be documented by research personnel.

### Interventions

**Figure 2:**
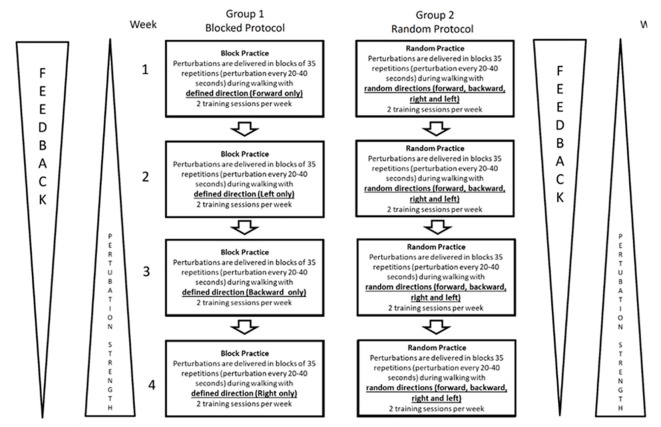
Details of the intervention training programs.

Block PBBT will be trained using a foretold magnitude and unidirectional perturbations during treadmill walking. The random PBBT will be trained using a computer-generated random multidirectional unexpected perturbation during treadmill walking. Each group will receive eight training sessions, twice a week for 4 weeks (Figure 2, Table 3, details of the training programs). Each session will last 20–30 min and will include 2 min of warm-up walking at participants’ preferred speed without any perturbations and 14–20 min of 35 unexpected perturbations while walking (based on the group motor learning technique). In the random PBBT, the perturbations will be given randomly every 20–40 s, and the training will end with a 2-min cool-down comfortable walking. The participants in the block PBBT group will be aware of the direction of the perturbation, given visual cues, and in each training session, only one perturbation direction will be trained. The goal of both training protocols (random PBBT and block PBBT) is mainly directed towards a cognitive understanding of the training and an improvement of self-confidence for exercises on higher perturbation magnitudes. Some general effects on muscle power and speed development, coordination, and conditioning (i.e., endurance) are expected. Everyone should progress to a higher perturbation magnitude (Figure 2, Table 3, the training programs).

**Table 2:** Details of the perturbation parameters that were used in the pre-testing and post-testing procedures.

Perturbation size	Displacement (cm)	Velocity (cm/s)	Acceleration (cm/s^2^)
Extra small	3	0.11	0.35
Small	6	0.22	0.7
Medium	9	0.44	1.5
Medium–large	12	0.66	2
Large	15	0.88	2.5
Extra-large	18	1.20	3

*Perturbations during the pre-testing and post-testing procedures will be induced through a mechatronic device that provides controlled and unexpected AP and ML platform translations during standing and walking. Four directional perturbations (right = R/left = L/forward = F/backward = B) in a random order of each of the six magnitudes, for a total of 24 perturbations*.

**Table 3:** Details of the perturbation protocols used in intervention training programs.

Training session	Platform displacement (cm)	Platform peak velocity (cm/s)	Platform peak acceleration (cm/s^2^)	Number of unannounced random* perturbations (per session)	Number of unannounced block* perturbations (per session)
1	1–3 cm	0.11 cm/s	0.35 cm/s^2^	35	35
2	2–4 cm	0.11 cm/s	0.35 cm/s^2^	35	35
3	3–5 cm	0.11 cm/s	0.35 cm/s^2^	35	35
4	4–6 cm	0.22 cm/s	0.7 cm/s^2^	35	35
5	5–7 cm	0.22 cm/s	0.7 cm/s^2^	35	35
6	6–8 cm	0.22 cm/s	0.7 cm/s^2^	35	35
7	7–9 cm	0.44 cm/s	1.5 cm/s^2^	35	35
8	8–10 cm	0.44 cm/s	1.5 cm/s^2^	35	35
9	9–11 cm	0.66 cm/s	2 cm/s^2^	35	35
10	10–12 cm	0.66 cm/s	2 cm/s^2^	35	35
11	11–13 cm	0.66 cm/s	2 cm/s^2^	35	35
12	12–14 cm	0.88 cm/s	2.5 cm/s^2^	35	35
13	13–15 cm	0.88 cm/s	2.5 cm/s^2^	35	35
14	14–16 cm	0.88 cm/s	2.5 cm/s^2^	35	35
15	15–17 cm	1.2 cm/s	3 cm/s^2^	35	35
16	16–18 cm	1.2 cm/s	3 cm/s^2^	35	35

*Random, perturbation direction forward/backward/left/right; Block, perturbation direction only forward or backward or left or right. The 16 training programs. Each session lasted 20–30 min and will include 2 min of warm-up walking in participants’ own preferred walking speed without any perturbations, 14–20 min of 36 unannounced perturbations while walking, given in random direction (right, left, forward and backwards) or block direction (one direction only per training session), and 3 min of cool down walking. The perturbation training program has 18 levels of difficulty with increasing levels of perturbations (i.e., increased displacement, velocity, and accelerations of the horizontal surface translations). During each session. Note: the listed platform translation unannounced perturbations were delivered in an unpredictable randomized sequence in the directions indicated forward, backward, left, and right for random PBBT group and for the block PBBT participants to one direction only in each training session.

**Figure 3:**
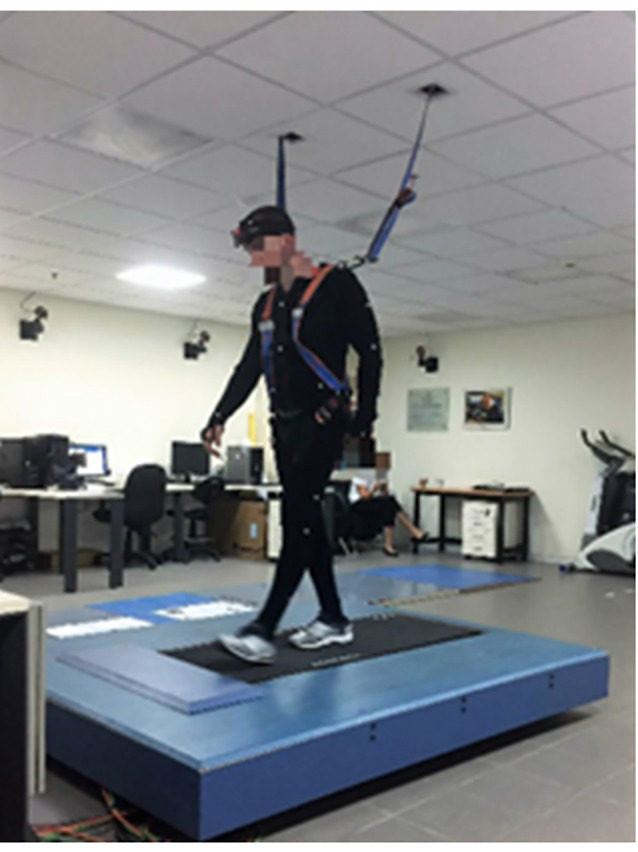
Photo of the BaMPer system. The system is composed of a motor-driven treadmill, mounted on a moving platform, with a motion controller, a safety harness, and an operator station (see text for more details).

We will use a perturbation treadmill (BaMPer system, see Figure 3) for providing controlled anterior–posterior (AP) and medial–lateral (ML) unannounced platform translations during treadmill walking. Both training protocols will be based on the principles of physical training and exercise prescription that include, e.g., awareness, continuity, motivation, overload, periodicity, progression, and especially the specificity principle. These important concepts are well established and accepted in the exercise physiology literature (Bhatt et al., 2012; Okubo et al., 2017; Gerards et al., 2017; Mansfield et al., 2017b; Sherrington et al., 2019). An exercise intervention targeting a certain function *must* provide a challenge/overload to the system and be progressive as well as specific to this function (Drowatzky and Drowatzky, 1999). The perturbation-based programs specifically target balance recovery reactions as follows:

#### Specificity

AP and ML unannounced balance loss, i.e., platform translations during treadmill walking in random order (random PBBT) or blocks (block PBBT), will be provided (Figure 2). In the random PBBT group, a software program was developed to provide random practice right/left/anterior/posterior surface translations, which was previously reported to ensure that the learned motor skill will generalize to a wide variety of situations and will optimize motor learning conditions (Drowatzky and Drowatzky, 1999; Dick et al., 2000). In the block PBBT group, anterior perturbations or posterior perturbations or right perturbations or left perturbations will be presented in separate blocks during the training session; in each session, the direction of perturbation will not be varied.

#### Overload and Progression

Sixteen protocols in increasing levels of perturbations will be used in the intervention training programs (see Table 3). The level of perturbation in the first training session will be personalized based on the following cutoff value and will be set according to the participant single-step threshold in standing. The single-step threshold is defined as the minimum perturbation magnitude that evoked a single-step response during the pre-testing procedure (i.e., overload).

The BaMPer system (Figure 3) is used to evoke perturbations during walking. The BaMPer is composed of a moving platform and a motor that provides unannounced surface translation perturbations in ML and AP directions during hands-free treadmill walking, aiming specifically to improve reactive balance reactions. The perturbations are programmed in the machine and are provided unexpectedly in terms of timing for both training methods (random PBBT and block PBBT). For example, in the first training session, the unannounced perturbations for both training groups (random PBBT and block PBBT) will vary with 1–2 cm displacement, 0.1–0.5 m/s velocity, and 0.5–3.0 m/s^2^ acceleration (see Table 3). However, while in the random PBBT, the perturbations were in random order, i.e., four directions (right/left/forward/backward), the perturbation in the block PBBT group in a single training session is to one direction only. In both training programs, a gradual increase in the difficulty of perturbation levels in terms of surface displacement, velocity, and acceleration will be made according to the trainee’s ability (see Figure 2, Table 3). The level of the perturbations that will be introduced in the next training sessions will be increased (i.e., progression) according to the trainee’s ability (i.e., individualization) and based on the following cutoff value: if a trainee did not experience falls after perturbation, i.e., were caught by the safety harness system during the training session, and feels that he/she can be further challenged; if not, the same level of perturbation magnitude will be introduced again until he/she successfully recovers balance in the entire session. During the training, the trainee will be instructed to avoid a fall. To reduce the potential fear of falling, the trainee will be encouraged to focus on increasing the speed and length of the compensatory stepping reaction (i.e., awareness). The trainee will be encouraged to try to regain normal walking with a minimum number of steps (i.e., choosing a single-step strategy rather than a multiple-step strategy, which is associated with increased risk of falling); they will be able to follow the initial step with as many additional steps as required to regain their balance.

#### Augmented Feedback

The content and scheduling of augmented verbal feedback from the trainer will be designed to assist in initial problem solving and then faded to avoid the possibility that they might interfere with motor learning as skill level progresses, according to the CPF (Guadagnoli and Lee, 2004). Mansfield et al. (2017a) found that concurrent augmented visual feedback reduced electrodermal level with practice, while no feedback did not, suggesting that feedback may help to reduce anxiety. In our study, participants in both groups will be provided information regarding two aspects of the stepping reaction. The first will be knowledge of the results of the stepping reaction (the occurrence of a fall into the harness or not) through information inherent in the task (the pull of the harness). The second will be knowledge of the performance of the stepping reaction (e.g., length of the step, direction, and strategy). Verbal feedback by the treating physical trainer will be additionally provided, for example, “steps need to be faster” or “try stepping further”; in addition, they will be instructed to “go back as fast as they can to their normal rhythm of walking after each perturbation.” During each week of training, feedback will be faded by the trainer and only feedback inherent to the task (fall into the harness or not) will be available for problem solving and learning.

The instructions to the participants in both groups will be “walk at your preferred walking speed and react naturally if you lose your balance.” The treadmill speed will be adjusted to the participant’s preferred speed. The perturbations will have 18 levels of difficulty with increasing levels of perturbations (i.e., increased displacement, velocity, and accelerations of the longitudinal translations; see Figure 2, Table 3, details of the perturbation protocols used in the training program). In both groups, the subject’s activities will be documented in each session. Assistance and support will be used for anyone who feels uncomfortable in the initial phase of the training. It is important to note that these exercises will be customized to each subject’s ability. They will be designed to be challenging but never dangerous.

### Data Collection and Outcome Measures

To assess the feasibility of the study, we will document the number of training sessions attended/missed, reasons for missed sessions, and the rate of missing data for the outcomes described below. In case the participant did not attend more than two consecutive training sessions or four training sessions in total, they will be excluded from the study.

After signing on the consent forms, the participants will complete a questionnaire at baseline that asks about their demographics and medical history and past falls. Participant’s balance reactions will be assessed during standing and walking. Participants will be exposed to right/left/forward/backward unannounced platform translations through a mechatronic device (i.e., BaMPer system; Shapiro and Melzer, 2010) that will be increased systematically and controlled in six perturbation magnitudes in increasing level of difficulty (see Table 2), for a total of 24 perturbation trials in standing: six right, six left, six forward, and six backward. During the walking trials, the participants will be exposed to 12 perturbations, six right and six left, since recovery step initiation in the forward and backward perturbation trials in walking is hard to explore.

During the examination, participants will wear a safety harness that prevents falls but does not otherwise restrict their balance recovery movements. Fall during the assessment session will be defined as load cell sensors detecting 30% or more bodyweight suspended by the safety harness. Participants will wear their own walking shoes and be instructed to react naturally to keep their balance and to prevent themselves from falling in response to perturbations. In case of falling into the harness system, grasping the examiner’s hand, or asking to stop the test by the subject, the examination will be stopped and will not continue to the next level. A seated rest break will be given whenever needed. The data will be analyzed observationally and kinematically for each trial.

### Step Thresholds in Standing

We will verify single-step threshold, multiple-step threshold, and fall threshold levels, for AP and ML directions, following a loss of balance using the Vicon Motion Analysis Systems (Oxford, UK), allowing image pauses, slow motion, and running of the image back and forth. The single-step threshold level is defined as the minimum perturbation magnitude that consistently elicited a single compensatory step for at least two consecutive perturbation magnitudes. The multiple-step threshold is defined as the minimum perturbation magnitude that consistently elicited a sequence of recovery steps, and the fall threshold is defined as the minimum perturbation magnitude that consistently elicited a fall into the harness system. These step threshold levels were shown previously to be independent predictors of a future fall (Hilliard et al., 2008; Carty et al., 2015).

### Kinematics of Reactive Stepping

Three-dimensional (3D) kinematic data will be collected through the optical motion capture (Vicon Motion Systems, Oxford, UK), providing kinematic analysis of a motion sequence. Sixteen infrared cameras covered the lab space, mounted at a height of 2.6 ± 0.2 m, and provide a capture volume of 5.5 × 1.2 × 2.0 m^3^ evenly scattered approximately 4 m around the treadmill. The cameras operate and sample simultaneously, at a frequency of 200 Hz, the location of 39 reflective markers placed on anatomical landmarks of the body and another two on the moving platform (Figure 3). The markers are attached to a prepared whole-body flexible suit, which comes in several sizes to properly fit each subject. Views from the 16 cameras are mapped onto a 3D coordinate system by the computer (Vicon System Software) using an internal direct linear transformation algorithm. All perturbations are digitized, transformed, and smoothed using a low-pass filter (Butterworth second-order forward and backward passes) with a cutoff frequency of 5 Hz. The Vicon System was shown to be valid and reliable. Overall trueness (systematic deviations) of a dynamic reference object was −0.23 ± 0.35 mm (−0.24 ± 0.36%), and overall uncertainty (random deviations) for dynamic measurements was 1.11 ± 0.94 mm (1.16 ± 0.99%). For lower-body assessment (10 cameras, foot region) during walking, the mean trueness and uncertainty were −0.08 and 0.33 mm, respectively (Eichelberger et al., 2016).

The following kinematic parameters of reactive stepping in standing will be extracted: (1) the step initiation duration in milliseconds is calculated as the time from surface horizontal translation to foot lift off the ground and the step initiative; (2) the first recovery stepping duration (ms) is calculated as the time from surface translation to foot contact on the ground, completing the step; (3) the first reactive step length is calculated as the Euclidian distance in centimeters that the ankle markers displaced from step initiation to first step recovery; and (4) the center of mass (CoM) path displacement is calculated as the Euclidian distance in centimeters that the CoM displaced from step initiation to first step recovery. We rely on the 39-marker model of Vicon Motion Systems for the CoM position; (5) margin of stability (MoS) AP is defined as the AP distance between the XCoM-AP and the anterior boundary of the BoS, defined by the leading toe marker (either RTOE or LTOE for the right and the left feet, respectively); and (6) MoS-ML is defined as the ML distance between the XCoM-ML and the lateral boundary of the BoS, defined by the ankle marker (right ankle and left ankle for the right and the left feet, respectively).

Using the method above, we found excellent interobserver reliability for single-step threshold, multiple-step threshold, first-step recovery initiation duration, step duration, and step length [intraclass correlation coefficient (ICC)2,1 = 0.917, ICC2,1 = 0.975, ICC2,1 = 0.978, and ICC2,1 = 0.918, respectively; *p* < 0.001; Batcir et al., 2018].

The following parameters will be used to quantify balance recovery during walking trials: (1) step initiation time (ms) will be calculated as the time from surface translation to the first ML deviation (right or left) of the marker placed on the stepping leg ankle joint, more than 4 mm from the average baseline after the surface translation; (2) first recovery stepping duration (ms) will be calculated as the time from surface translation to foot contact on the ground; (3) first compensatory step length will be calculated as the Euclidian distance in centimeters that the ankle markers displaced from step initiation to first step recovery, i.e., foot contact; (4) first swing phase duration (ms) will be calculated from the step initiation time to when the foot contacted the ground, completing the first recovery step; and (5) the estimated distance of the CoM from the BoS (dBoS) is defined as the distance in ML direction (cm) of the estimated CoM (eCoM) from the theoretical edge of the BoS provided by the feet (i.e., the ankle marker) at the point of step initiation. A larger dBoS reflects a mechanically unstable condition at the point of first step initiation; i.e., the vertical projection of the eCoM at step initiation will be larger with respect to the BoS.

### Voluntary Step Execution Test

To assess the proactive balance function, participants will stand on a single force platform and will be instructed to voluntary step as quickly as possible following a somatosensory cue, given randomly on one of their feet as described in detail in previous articles (Melzer and Oddsson, 2004; Melzer et al., 2007b; Batcir et al., 2018). A total of six trials will be conducted in single-task (ST) conditions, as well as in dual-task (DT) conditions, while performing the Stroop test. The temporal events will be extracted from the step execution data: (a) reaction time; (b) foot contact time; and (c) preparation time (Melzer and Oddsson, 2004; Melzer et al., 2007b). The ICC values for intratester reliability, for older adults, are good to excellent in these step parameters across ST and DT task conditions (0.62–0.88; Melzer and Oddsson, 2004; Melzer et al., 2007c). A quick execution of a step is an early line of defense to avoid falling, which may be considered the most important postural reaction to prevent a fall (Maki et al., 1994; McIlroy and Maki, 1999; Melzer and Oddsson, 2004; Melzer et al., 2007b). We previously found that the step execution test is sensitive to the effects of age in both ST and DT conditions, (Melzer and Oddsson, 2004) identified older adults who reported retrospectively a fall under the dual-task condition but not in single-task condition, (Melzer et al., 2007b) and predicted future falls with no added value to dual- over single-task condition (Melzer et al., 2010b); however, we found that the dual-task paradigm of the voluntary step execution test was able to detect the probability of being seriously injured from a fall (Melzer et al., 2009). In a recent meta-analysis of 54 studies (*n* = 8, 385), Okubo et al. (2020) showed that stepping performance was significantly worse in fallers compared to nonfallers (Cohen’s *d* 0.55, 95% CI 0.48–0.66, *p* < 0.001, *I*^2^ 68%). This was the case for both volitional and reactive step tests. Twenty-two studies (*n* = 3, 503) were included in a diagnostic meta-analysis that showed that step tests have moderate sensitivity (0.70, 95% CI 0.61–0.77), specificity (0.69, 95% CI 0.61–0.77), and area under the receiver operating characteristics (AUROC; 0.76, 95% CI 0.67–0.83) in discriminating fallers from nonfallers.

#### Postural Stability Test and Stabilogram Diffusion Analysis

The participants will be instructed to stand barefoot as still as possible on a force platform in a standardized stance, with their feet close together. There were four 30-s quiet-standing trials with eyes open (EO) and four trials with eyes close (EC) and blindfolded. The center of pressure (CoP) and ground reaction force data will be collected with the Kistler 9287 force platform. Evaluation of balance control will be made using the traditional measure of postural sway in both EO and EC conditions (e.g., ML sway, AP sway, mean sway velocity, and mean sway area). We will also calculate the stabilogram diffusion analysis (SDA) parameters from CoP data [e.g., critical displacement (Cd) and critical time (Ct)]. The transition point between the short-term and long-term behaviors has been termed the Ct, and sway displacement has been termed the Cd at which closed-loop control begins to dominate sway behavior. It was described in detail by Collins and De Luca (Collins and De Luca, 1993; Collins et al., 1995). The ICCs for the CoP sway parameters are excellent in the EC condition: ML sway (ICC = 0.933), AP sway (ICC = 0.946), sway area (ICC = 0.710), sway length (ICC = 0.945; Bauer et al., 2008) The ICC showed fair to good reliability in the SDA parameters: Ds (ICC = 0.79), Dl (ICC = 0.50), Cd (ICC = 0.66), and Ct (ICC = 0.63; Chiari et al., 2000). The postural stability and SDA method have been adopted by several research groups who have shown that the parameters of postural stability and SDA are sensitive to the effects of age (Melzer et al., 2001) old adults who retrospectively reported falls (Melzer et al., 2004, 2010a) and older adults who reported injury as a result of a fall (Kurz et al., 2013).

Also, clinical measurements and questionnaires will be conducted:

(1) The Berg Balance Scale (Berg et al., 1989) assesses balance and fall risk with an excellent interrater (ICC = 0.98) and intrarater (ICC = 0.99) reliability.
(2) Late-Life Function and Disability Instrument (Melzer et al., 2007a) is a self-reported function that measures difficulty in performing basic and advanced daily physical tasks with ICCs of 0.91–0.98 for the function component.
(3) Short Falls Efficacy Scale-International (Yardley et al., 2005) evaluates fear of falling while performing indoor and outdoor daily activities with an excellent ICC of 0.83.
(4) MMSE’s (Folstein et al., 1975) test–retest reliability was excellent (Pearson *r* = 0.98, *p* < 0.001), and its validity was found to be high and significant as well (*r* = 0.77, *p* < 0.0001; *r* = 0.66, *p* < 0.001, for the verbal and performance subscores, respectively).

#### Power and Sample Size Calculation

Since it is well established that perturbation training is effective in improving balance and reducing falls (Sherrington et al., 2019), the primary purpose of this study is to determine the effect of block PBBT on major components of reactive balance control among older adults: fall threshold and multiple-step threshold. Our primary hypothesis is that improvements in reactive balance control following block PBBT will be not inferior to the improvements following random PBBT. We will use the non-inferiority approach. Non-inferiority trials examine whether a new experimental treatment (i.e., block PBBT) is not unacceptably less efficacious than another treatment (i.e., random PBBT) already in use. Our primary outcome measures are the multiple-step threshold and fall threshold that are rarely used as outcome measures although multiple-step threshold levels are shown previously to be independent predictors of a future fall (Hilliard et al., 2008; Eichelberger et al., 2016) and the fall threshold is a novel measure of balance recovery function that seems to be ecologically valid. Both the fall threshold and multiple-step threshold were used as primary outcome measures in our recent study (Handelzalts et al., 2019) where we found that 11 of 11 stroke patients (100%) who participated in the random PBBT intervention improved their fall threshold (from 4.5 ± 2.0 to 6.5 ± 1.3) compared with only four out of 13 stroke patients who participated in the nonperturbation training intervention and that the multiple-step threshold to forward and backward perturbations was improved (from 1.9 ± 1.4 to 3.8 ± 1.7 and from 2.6 ± 1.4 to 4.5 ± 2.1, respectively). The sample size for a non-inferiority trial with a fall threshold and multiple-step threshold was calculated using the formula below (Statistical Solutions Ltd., 2020; nQuery 8—Power Sample Size for Group Sequential Trials version 8.6.1.0).

Sample sizes for each of the primary outcomes are outlined in Table 4. In regard to the fall threshold and the multiple-step threshold (backward), the sample size that was calculated for each group is similar, *n* = 19. A two-group one-sided 0.05 significance level *t*-test will have 80% power to reject the null hypothesis that the block PBBT and random PBBT are not noninferior [the difference in means of the fall threshold and multiple-step threshold (backward), μR − μB, is 2.00 and 1.90, respectively, or farther from zero in the same direction), in favor of the alternative hypothesis that the means of the two groups, i.e., block PBBT and random PBBT, are noninferior, assuming that the expected difference in means is 0.00 and the common standard deviation for the fall threshold is 2.40 and the common standard deviation is 2.00 for the multiple-step threshold (backward; based on an earlier study; Handelzalts et al., 2019). In regard to the multiple-step threshold (forward), the calculated sample size in each group is 8, and a two-group one-sided 0.05 significance level *t*-test will have 80% power to reject the null hypothesis that the block PBBT and random PBBT are not noninferior (the difference in means, μR − μB, is 1.90 or farther from zero in the same direction), in favor of the alternative hypothesis that the means of the two groups are noninferior, assuming that the expected difference in means is 0.00 and the common standard deviation is 1.40 (Table 2). To account for attrition rates reported to be about 25% involving training in older adults, (McMurdo et al., 2000) it was decided to include 25 participants in each group (19 × 1.25 = ~25) for a total of 50 participants.

**Table 4:** Sample size estimation.

	Fall threshold	Multiple-step threshold (forward)	Multiple-step threshold (backward)
Test significance level α (one-sided)	0.05	0.05	0.05
Non-inferiority limit difference, Δ0	2.00	1.9	1.90
Expected difference, Δ1	0.00	0	0.00
Δ0 − Δ1	2.00	1.90	1.90
Common standard deviation, σ	2.40	1.4	2.0
Effect size, δ = |Δ0 − Δ1|/σ	0.833	1.357	0.826
Power, %	80	80	80
Sample size, per group, *n*	19	8	19

### Statistical Analysis

Cohort descriptors and baseline values for primary and secondary outcomes will be compared between groups using Mann–Whitney *U* tests (continuous variables) or Fisher’s exact test (categorical variables). Measures that differ between groups at baseline may be used as covariates in the analysis of primary and secondary outcomes. To test the primary hypothesis, we will calculate the 95% CI for the post-intervention difference between groups (block PBBT minus random PBBT) for the primary outcomes [fall threshold and multiple-step threshold (backward and forward)]. Our hypothesis of non-inferiority will be supported if the lower limits of the 95% CIs are greater than the negative value of the non-inferiority limits (Schumi and Wittes, 2011). We will use both intention-to-treat and per-protocol analyses to test the primary hypothesis, as is recommended (Schumi and Wittes, 2011); per-protocol analysis will include only those participants who completed at least 80% of the training sessions. We will also use paired *t*-tests for each group individually to determine if the groups improve over time in the primary and secondary outcomes. To address the secondary objectives, we use analysis of covariance (ANCOVA) to compare the posttraining voluntary step execution test and postural sway parameters as well as the Berg Balance Score, Late-Life Function and Disability Index, and FES-I scores between groups, with the baseline value for each measure as a covariate. The dependent variable will be rank-transformed prior to conducting the ANCOVA to allow for nonparametric analysis.

### Adverse Events

Based on our previous studies, very mild adverse events related to PBBT have been reported, i.e., delayed-onset muscle soreness, fatigue, or exacerbation of joint pain in older adults (McMurdo et al., 2000; Kurz et al., 2016) and even in stroke patients (Handelzalts et al., 2019) with a similar frequency and severity of adverse events for both the PBBT and control groups, who completed more “traditional” balance training. Medical attention was not required for those who participated in the PBBT.

### Safety

Since the assessment and intervention are challenging for balance control, there is a small risk that older adults will lose his/her balance, especially during the balance recovery assessment. For that matter, we use a safety harness attached to a secure point overhead that will be worn for all postural perturbations to prevent a fall. Additionally, the research assistant or physiotherapist can provide assistance to prevent a fall. A similar harness system will be used during the interventions to ensure patient safety during the training session (see Table 2). In addition, training will be administered by a trained physiotherapy student that will tailor the training to the patient’s abilities. Assessments will be completed by a trained research assistant with a health sciences background. We have administered tens of thousands of postural perturbations with more than 100 older adults and about 40 stroke survivors with varying balance abilities in our previous research studies, and no one suffered an injury as a result of an induced balance perturbation. Also, in the cases where the participants were caught by the safety harness system or researcher, they did not suffer a physical injury. In case of a physical injury, the physiotherapist will provide first aid, will advise the participant regarding follow-up with medical aid, and will follow up with the participant after a day or so.

### Data Monitoring Committee

A data monitoring committee is not required for this study since the PBBT is a low-risk intervention for older adults. Adverse events that meet all three of the following criteria will be reported immediately to the institution’s research ethics board, as is routine practice: (1) unexpected in terms of nature, severity, or frequency; (2) related or possibly related to participation in the research; and (3) suggestion of a potential increase in risk of harm to research participants or others. All adverse events will be collated and evaluated biannually by the principal investigator.

### Trial Status

The study is currently recruiting participants. Enrollment began on February 1, 2019. We will complete the recruitment, training, and T1 and T2 data collection by March 1, 2021. We will complete the data analysis by December 31, 2021.

## Discussion

### Strengths

The perturbation training is not a novel intervention method, and the technology that provides unexpected perturbation during the treadmill walking method to improve balance function was used previously. The training methods presented here (random PBBT vs. block PBBT) are specific and incorporated progressive overloading and individualization. However, as far as we know, the comparison between random and block perturbation training methods to improve reactive balance was never examined in older adults. Our research project will investigate which training protocol will show generalization (positive transfer) or interference (negative transfer; random PBBT vs. block PBBT). Additionally, it will define if reactive balance control following block PBBT will not be inferior to the improvements following random PBBT.

This research will also address a key question about the generalizability of balance training and the underlying locomotor plasticity in older adults using gait perturbation as an innovative approach to improve balance reactive responses also in standing. The intervention will add physical activity to community-dwelling older adults. Therefore, our findings will be most likely improving their function and be directly relevant to active and healthy aging. We believe that the implantation of the best paradigm for training protocol into clinical interventions will reduce the incidence of falls in a highly efficient and cost-effective manner, especially for older adults. If we will find the benefits of random PBBT compared with block PBBT or vice versa, this would allow therapists and patients to more easily use a more customized fall-prevention training in their regular practice.

We are motivated by the work done recently by Takazono et al. (2020) which has explored the effects of the block and the random perturbation training on the stability of compensatory arm and leg movements (using the CALM scale; de Souza et al., 2019) in young participants. They found better generalization and retention performances in the random training group. Those results support our hypothesis and emphasize the need to explore those training schedules with older adults. The proposed amount of training in the block and random PBBTs is similar and exceeds that of previous studies of perturbation training in older adults.

### Limitations

There is a risk that both random PBBT and block PBBT methods introduced here might be difficult at the beginning of the training; thus, muscle soreness will occur. Also, there is a risk of balance loss and fall; however, we will use a harness system to ensure that a fall will not occur. Since PBBT is a challenging training approach, there is a risk that older adults will stop participating in the program (dropout). Because the outcome measures in this study are related to balance function and risk for falls, it is unlikely that our results will be definitive regarding the ability of these PBBTs to translate to a reduced number of falls and injurious falls among older adults. We will monitor real-life falls in a 1-year period post-intervention, using a diary and monthly telephone calls, but the sample size for such analysis will be too small. However, we assume that due to the low sample and relatively fit older adults, the improvement in balance function will not carry over to real-life falls. Our study will address relatively healthy independent older adults; therefore, the results could not be implemented on frail elderly or other populations. Also, since the number of participants is small, the study has a pilot character. Also, data analysis will address the following question: what is the optimal sample size for the primary outcome parameter, which will be used to estimate the sample size for the larger trial. Finally, although the assessor and the participants in this trial are blinded, the therapist delivering the intervention cannot be blinded to intervention allocation, which potentially introduces a source of bias.

## Ethics Statement

The studies involving human participants were reviewed and approved by Soroka Medical center approval #0396-16-SOR. The patients/participants provided their written informed consent to participate in this study.

## Author Contributions

IM conceived the study and is the grant holder. IM, HN, and IP developed the intervention. IM, HN, IP, MS, and IS contributed to the study design and drafted the manuscript. All authors contributed to the article and approved the submitted version.

## Conflict of Interest

IM owns a patent on some of the technology used in the perturbation system. The remaining authors declare that the research was conducted in the absence of any commercial or financial relationships that could be construed as a potential conflict of interest.
